# BioTarget: A Computational Framework Identifying Cancer Type Specific Transcriptional Targets of Immune Response Pathways

**DOI:** 10.1038/s41598-019-45304-x

**Published:** 2019-06-21

**Authors:** Tham H. Hoang, Yue Zhao, Yiu Lam, Stephanie Piekos, Yueh-Chiang Han, Cameron Reilly, Pujan Joshi, Seung-Hyun Hong, Chang Ohk Sung, Charles Giardina, Dong-Guk Shin

**Affiliations:** 1 University of Connecticut, Department of Computer Science and Engineering, Storrs, CT 06269 USA; 2 University of Connecticut, Department of Pharmaceutical Sciences, Storrs, CT 06269 USA; 3 University of Connecticut, Department of Molecular and Cell Biology, Storrs, CT 06269 USA; 4 University of Ulsan College of Medicine, Department of Pathology, Asan Medical Center, Seoul, Republic of Korea

**Keywords:** Computational models, Data integration

## Abstract

Transcriptome data can provide information on signaling pathways active in cancers, but new computational tools are needed to more accurately quantify pathway activity and identify tissue-specific pathway features. We developed a computational method called “BioTarget” that incorporates ChIP-seq data into cellular pathway analysis. This tool relates the expression of transcription factor TF target genes (based on ChIP-seq data) with the status of upstream signaling components for an accurate quantification of pathway activity. This analysis also reveals TF targets expressed in specific contexts/tissues. We applied BioTarget to assess the activity of TBX21 and GATA3 pathways in cancers. TBX21 and GATA3 are TF regulators that control the differentiation of T cells into Th1 and Th2 helper cells that mediate cell-based and humoral immune responses, respectively. Since tumor immune responses can impact cancer progression, the significance of our pathway scores should be revealed by effective patient stratification. We found that low Th1/Th2 activity ratios were associated with a significantly poorer survival of stomach and breast cancer patients, whereas an unbalanced Th1/Th2 response was correlated with poorer survival of colon cancer patients. Lung adenocarcinoma and lung squamous cell carcinoma patients had the lowest survival rates when both Th1 and Th2 responses were high. Our method also identified context-specific target genes for TBX21 and GATA3. Applying the BioTarget tool to BCL6, a TF associated with germinal center lymphocytes, we observed that patients with an active BCL6 pathway had significantly improved survival for breast, colon, and stomach cancer. Our findings support the effectiveness of the BioTarget tool for transcriptome analysis and point to interesting associations between some immune-response pathways and cancer progression.

## Introduction

A common approach for interpreting RNA-seq data is to assess the expression level of genes along a curated signaling pathway, and then “score” the pathway for its potential level of activity. The methods to score pathways have steadily evolved since the arrival of the first high-throughput expression technology “DNA microarray” in the 80s. Khatri *et al*.^1^ summarizes three generations of pathway scoring methods: the 1st generation Over-Representation Analysis (ORA) Approaches, the 2nd generation Functional Class Scoring (FCS) Approaches, and the 3rd generation Pathway Topology (PT)-Based Approaches. Example systems of each generation are, respectively, GOstat^2^, GSEA^3^ and SPIA^4^. Notable in the 3rd generation is the use of pathway topology in generating the scores. In PT, prior knowledge of activation and suppression relationships captured in the gene/protein network is used to improve score estimation compared to the previous generation systems that merely used frequency of differentially expressing genes for the calculation, e.g., Fisher’s Exact test in GOstat and Kolmogorov-Smirnov statistic in GSEA. PT methods themselves evolved as different types of omics datasets become available. Isik *et al*.^5^ proposed a hybrid method in which both transcriptome and ChIP-seq data are combined to estimate activation/suppression levels of signaling networks. PARADIGM^6^ combines mutation and gene expression data using factor graph, to better assess pathway perturbation obtainable from cancer samples. APA^7^ aims to detect altered pathways by dynamically calculating pathway rewiring through analyzing correlation between genes, but this system does not use prior knowledge. Most recently, we reported our own PT methods which aim at identifying “pathway routes” as perturbed portion using a Bayesian model built from topological pathways^8,9^.

What we report here goes beyond the aforementioned pathway scoring systems. Our goal is to extend “curated” pathways downstream of a transcription factor (TF) involved in a signaling network, i.e., TF target genes. This study’s objective stems from the observation that majority of existing signaling pathways are sparse in cataloging the events occurring inside nucleus where TFs bind DNA to regulate mRNA transcription. Our hypothesis is that these TF regulatory events are highly context-sensitive, meaning the regulatory events may heavily depend on the temporal and spatial context of the experimental condition. As such, the field has not tackled this problem yet. However, we conjecture that the recent availability of ChIP-seq data through ENCODE and other large scale genomics initiatives like TCGA offer opportunities to incorporate TF target genes into the pathway analysis.

**Figure 1:**
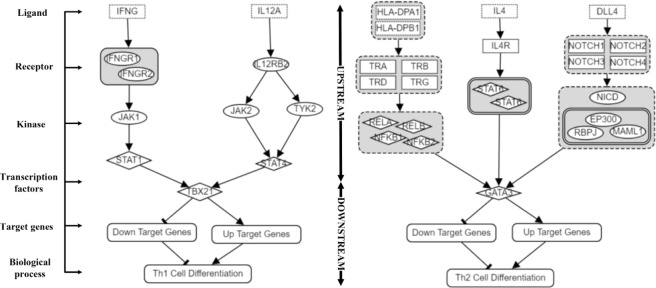
Molecular pathways of Th1 and Th2 Cell Differentiation are modeled into two parts: Upstream and Downstream of transcription factor (TF), in this case, TBX21 and GATA3, respectively. The model also categorizes the TF target genes into two types - Up targets and Down targets.

Figure 1 illustrates our methodology for extending the curated pathways. This figure shows signaling activities that may typically occur during an immune reaction in which T cells differentiate into Th1 or Th2 helper cells. Here TBX21 and GATA3 are two master TF regulators that mediate the cell-based and humoral immune responses, respectively. In Th1 cell differentiation, cell differentiation signaling starts from the ligand, IFNG, to the TF, TBX21, and alternatively from IL12A to TBX21. We posit that attempting to estimate T cell progression through Th1 differentiation may not be determinable only by examining the upper portion of the pathway; the downstream activity of TBX21 should also be examined. Unfortunately, as this limitation has been suggested, the literature is scarce in documenting such events. The same argument is applied to the differentiation of Th2. If the downstream events of TBX21 or GATA3 are well curated in both pathways, then pathway scoring can pinpoint with a higher accuracy if T cells are heading toward Th1 or Th2 helper cells.

There are a number of issues to be dealt with when attempting to extend pathways. The first issue is whether some pathways are more amenable to the type of TF downstream extension. Our choice is that immune signaling pathways are good candidates because of the established consensus on these immune pathways. The second issue is which transcriptome datasets is more appropriate to use for the pathway extension. As stated earlier, TF downstream identification can be context-sensitive and, as such, a sufficiently large number of “birds of a feather” transcriptome datasets should be used for a cohort-based analysis. The public TCGA datasets are good candidates since each cancer type can establish a context. The third issue is if there is any quantifiable measure that can guide the actual extension process. For example, if one gene *G*_*i*_ is concluded to be a target of the TF *T*_*j*_, then how to gauge the impact of adding the relationship “*T*_*j*_ regulates *G*_*i*_” into the pathway? We consider that the Kaplan-Meier (KM) survival analysis^10^, the non-parametric statistical method used to study the efficacy of treatments or conditions of cancer patients, can be used for developing such assessment method.

Lastly, one will also have to show the impact of extending the pathway. When using TCGA datasets, the question can be answered by testing if our method can meaningfully stratify patients. For this evaluation, KM survival analysis can also be used, since the relationship between immune responses and cancer progression has been studied extensively^11,12^. Indeed, our analysis outcomes show that the scores obtained using extended pathways effectively stratify patients into groups with different survival characteristics, supporting the value of the scoring system.

## Results

**Figure 2:**
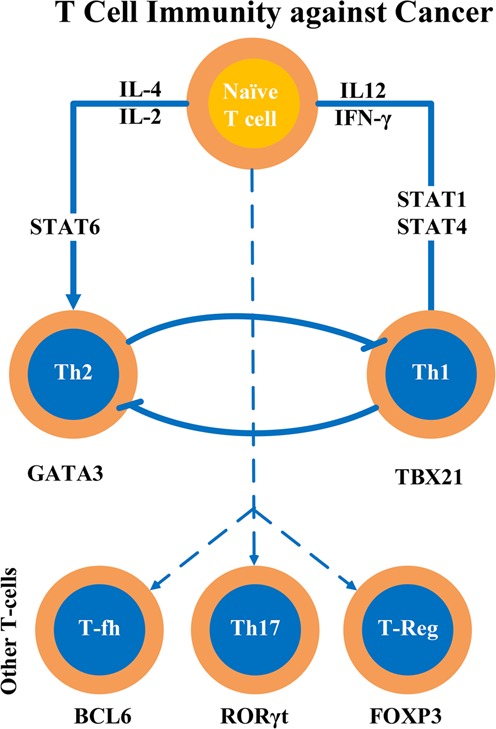
T cells are differentiated into multiple T cell subtypes including Th1, Th2, T-fh, Th17 and T-reg cells. Each cellular differentiation pathway is known to be controlled by key transcription factors. Examples include TBX21, GATA3, BCL6, ROR*γ*t, and FOXP3 for Th1, Th2, T-fh, Th17, and T-reg cells, respectively. Th1 cells and Th2 cells interact with each other to balance immune responses to cancer.

Prior to presenting the computational analysis outcomes, some background of T cell differentiation is given. Figure 2 shows the cellular level depiction of how naïve T cells are differentiated into five different T cell subtypes, Th1, Th2, T-fh, Th17 and T-reg cells, among many other subtypes known in the literature. How the naïve T cells differentiate into each subtype is “partly” known and such process is often summarized in molecular pathway diagrams as shown in Fig. 1. Transcriptome analysis of cancer tissue will help scientists discover to what degree differentiation into any of these subtypes is occurring in the cancer tissue. Scientists would like to use this information to infer disease etiology. For example, an effective immune response to cancer cells can be seen controlling lesion growth and progression. However, inflammatory signaling by tumor-associated immune cells can also be cancer promoting. According to Wang *et al*.^13^, if the immune response is shifted towards Th2-dominance, lesion progression is favored. What we report here is how the three pathways known for regulating Th1, Th2 and T-fh cell differentiation can be extended and what the implications of these extensions are.

### Extending Th1 cell differentiation pathway with cancer type specific target

In this experiment, we processed ENCODE ChIP-seq data to produce 1275 potential target genes of TBX21 using the method described in Section 4.1 (i.e., genes having Gaussian peaks 2000 bp near the transcription start sites). These potential target genes are input into the BioTarget pipeline, which was run for each of the five TCGA cancer datasets, Stomach Adenocarcinoma (STAD), Breast Invasive Carcinoma (BRCA), Colon Adenocarcinoma (COAD), Lung Adenocarcinoma (LUAD), and Lung Squamous Cell Carcinoma (LUSC). Table 1 shows the identified target genes of TBX21 categorized into two groups, Up and Down. Multiple facts are noticeable. First, many identified targets have already been reported in immunology or cancer literature as they have been annotated with corresponding citations. Second, there are many target genes commonly found in the same direction of multiple cancer cohorts. For example, CD48, also known as B-lymphocyte activation marker, as Up target in four out of the five cancer cases. Similarly, interleukin 21 receptor, IL21R, is included as Up target in STAD, COAD, and LUSC. Third, genes are uniquely identified as either up or down regulated (i.e., none of the genes identified as the Up target appears as the Down target in any case)). Fourth, genes not previously implicated in the immune system or cancer are discovered and reported in the table. For example, in COAD case, NKG7 and TBC1D10C are included in the Up target list, but neither of these have been cited in the immune/cancer literature, nor in curated annotation resources such GeneRef, GeneOntology, and IHOP, to the best of our knowledge. Collectively, our method reports a total of 17 genes as Down targets of TBX21 for STAD, which are unknown in immunology or cancer literature. Lastly, we note that even if a citation is associated with many listed genes, the citations may not necessarily discuss the relationship between the cited gene and TBX21 in the context of the specific cancer type that the analysis was performed.

**Table 1:** Target genes of transcription factor TBX21 identified in Th1 Cell Differentiation pathway.

Study	Up/Down-regulated genes and their functionality
STAD	Up: APOBEC3H^33^, ARHGAP30^34^, CCL4^35^, CD48^36^, CD53^36^, CORO1A^37^, CRTAM^38^, CXCR3^39^, FCRL6^40^ GPR171^41^, GPR65^42^, GRAP2, GZMK^43^, HCLS1^44^, HCST^45^, IFNG^46^, IL18RAP^47^, IL21R^48^, ITGAL^47^, KLHL6, LAX1, LSP1, LY9, NKG7, P2RY10, PDCD1LG2^49^, SLAMF8^36^, SLFN12L, TBC1D10C, TRIM22 Down: CDCA7L^50^, COG3, CSNK2A1, CSTF1, F11R, FARSB, FLAD1, GSTA4, HUS1, NLK, PIGV, SLC3A2, SPATA17^51^, TAF2, TBRG4, TSTD1, ZDHHC6
BRCA	Up: AGAP2, CD28^36^, CD48^36^, CD53^36^, FCRL3^52^, FCRL6^53^, GPR171^41^, GPR183, GRAP2^52^, HCLS1, IL18RAP^47^, IL23R, LAX1, LRMP, LY9, MS4A1^54^, NKG7^55^, PDCD1LG2, SLC9A9^56^, SLFN12L, SRGN, TBC1D10C Down: ARFIP2, BTRC, COPB1^57^, KBTBD4, SPATA17^51^, TSTD1, TTC26^58^
COAD	Up: IL21R^48^, ITGAL^47^, NKG7, TBC1D10C Down: AARSD1, COX17, F11R, LYSMD1, MACC1, MRPL9, PON2, RNF43, RPL12, RPL14, RPL23, RPL5, RPS18, SMG7, SNRPE, TARS2, ZNF774
LUAD	Up: ARHGAP30^34^, CCL4^35^, CD48^36^, CORO1A^37^, CRTAM, CXCR3, FCRL3, FCRL6^40^, GRAP2^59^, GZMK, IFNG^46^, IL18RAP^47^, ITGAL^36^, NKG7, TBC1D10C Down: BYSL, COPB1^57^, DHRS13, ELL3, GSPT1, ILF2, LYSMD1, MRPS18B, NDUFS1, PEMT, PEX13, PPP1R11, PTS, SEC. 23B, SLC35B2, SLC39A7, SNRPE, TSG101, TSTD1, VPS52
LUSC	Up: ARHGAP30, CCL4, CD28^36^, CD48^36^, CD53^36^, CD86^36^, CIITA, CORO1A^37^, CRTAM, CTSS, CXCR3^39^, FCRL3, FCRL6^40^, GPR171^41^, GPR65, GPSM3, GRAP2, GZMK, HCST, IL18RAP^47^, IL21R^48^, ITGAL^47^, ITGB7, LAIR1, MNDA, NKG7, P2RY10, PIK3CG, PTPN22, SELPLG, SLAMF8^36^, SLC15A3, TBC1D10C, ZBP1, ZC3H12D Down: ACTL6A, COPS2, DDX18, ERAL1, GSTA4, METTL2A, MRPL30, PAK1IP1, PDCD10, PHF5A, SLC35F5

**Figure 3:**
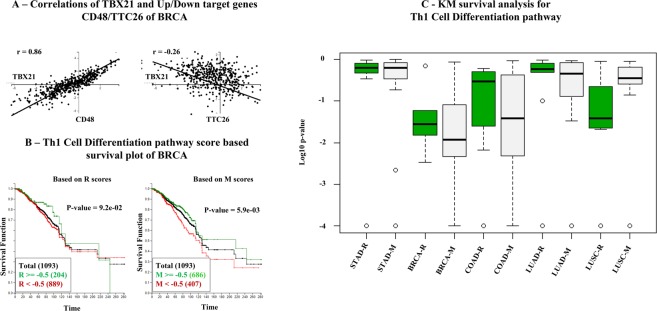
(**A**) Correlation of transcription factor TBX21 and CD48/TTC26 as Up/Down targets of TBX21 identified by our approach in breast cancer cohort. (**B**) BRCA breast cancer cohort survival analyses for the extended Th1 Cell Differentiation pathway. (**C**) KM Analyses conduced on five TCGA cancer cohorts for the extended Th1 Cell Differentiation pathway.

Next, we performed two types of post analysis, a correlation study to check the quality of the discovered targets and the KM-survival analysis to study the impact of extended pathways with newly added TF target genes. Figure 3A shows two correlation graphs. The first one shows that the transcriptome signals obtained from TBX21 are highly correlated with downstream signal of the Up gene CD48 (*r* = 0.86) in the breast cancer case (*n* = 1093). The second graph shows that the gene expression levels of TBX21 and its BioTarget identified Down gene TTC26 are negatively correlated (r = −0.26) in the breast cancer case. An additional ten correlation graphs are given later in Supplementary Information in order to document the high correlation between TBX21 and representative Up/Down targets identified in each of all five cancer cases.

Kaplan-Meier (KM) survival analysis is used here to evaluate what the extended pathway can do, i.e., can the two subpopulations of a cancer patient cohort partitioned by some threshold pathway score improve patient prognosis when extended targets are used? Figure 3B contrasts two cases of the KM analysis performed on the breast cancer cohort (BRCA) with two different pathway scoring methods obtained for the Th1 Differential pathway, one using only the upstream of TBX21 signals (referred to as the “R-score”) and the other using TBX21 and its identified downstream target genes (referred to as the “M-score”). In this example, the threshold pathway score used for the split are R = −0.5 and M = −0.5 as shown in Fig. 3B. This comparison demonstrates a clear improvement in patient stratification. The KM analysis p-value is significantly reduced (p-value = 0.0059) when the augmented Up/Down TBX21 targets are taken into account. One known issue of the KM analysis is its sensitivity to the choice of threshold values used to divide the cohort. For this reason, multiple comparisons are done with varying threshold values. Figure 3C shows all KM p-values produced by varying thresholds from −1 to 1 for both R and M scores. It shows the distribution of p-values using a pair of box plots for each cancer type, one for R-score based (colored in green) and one for M-score based (colored in gray), e.g., STAD-R and STAD-M for STAD. Among the five cohort cases, the survival analysis p-values improved in three cases with its best improvement notable for the COAD case.

### Extending Th2 cell differentiation pathway with cancer type specific target genes

**Figure 4:**
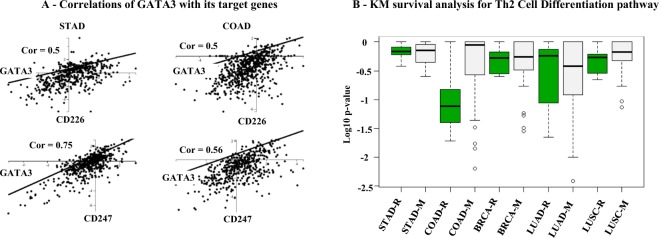
Cohort-based study for the extended Th2 Cell Differentiation pathway.

This time we processed ENCODE ChIP-Seq binding profiles of GATA3 to identify 355 target genes (c.f., Section 4.1). These genes are input to the BioTarget pipeline five times, one for each of the five cancer cohorts. An absolute thresholding scheme was applied, i.e., −0.3/0.3 for negative/positive correlation between TF and its target gene, for target derivation. Target genes of TF GATA3 are reported in Table 2. Figure 4A illustrates high correlation between GATA3 and its potential target genes CD226 and CD247 discovered by the pipeline, respectively, for STAD and COAD. Figure 4B shows the outcomes of the KM analysis performed with varying pathway score threshold values for the entire five cohorts. Some improvement is shown for the case of LUAD, but indistinguishable in the cases of STAD and BRCA and it became worse in the cases of COAD and LUSC.

**Table 2:** Target genes of transcription factor GATA3 identified in Th2 Cell Differentiation pathway.

Study	Up/Down genes and their functionality
STAD	Up: CAMK2D, CD226^60^, CD247^36^, GNA15^61^, HIVEP2, IKZF3, IL18R1^47^, IL2RB^47^, ITK, MAP3K5, TRAD Down: PRKCH, RASGRP1, RASSF5, SEMA4D^62^, SMAP2, SPEF
BRCA	Up: ARHGAP26^63^, ARV1, ASB2, ATXN10^64^, AXIN2^65^, BCAT2, BLCAP, CAMK2D, CBLB, CCDC12, CD226, CD247^36^, CDK5RAP2, CHEK2, CHL1, CRB2, CX3CR1, EIF3D, ETV5, FAM124B, FAM3C, FOXP1, GNA15^61^, HINT1, HSCB, IKZF3, IL13, IL18R1, IL2RB^66^, IRF9, ITK, LSM4, MAP3K5, MED29, MOBP, MYL12B, MYL6B, NHSL2, NOL3, NSMCE1, POLR3GL, PPP3CA, RASGRP4, RASSF5, ST8SIA1, STK40, TCF3, TGFBR3, TMEM131, TMEM134, TRADD, TULP2, UNKL, XIRP1, ZDHHC24, ZFP36, ZNRF Down: ZFPM1, AEBP2, AGPAT5^67^, ANK1, ASAP1, ATN1, BZRAP1, CCDC146, CCDC88C, CCR6, CDK8, CEP68, CHD3, CISH, CRB3, FANCC, FBXL8, HIVEP2, HSF4 IFT20, IGF1R LRRC6, LYRM7, MAP2K4, MAPK6, MAPKAPK3, PRKCH, RAD50, RALGAPB, RASGRP1, NUCB1, PAF1, PAQR8, PDE3B, PELI1, RNF31, RORA, RPS6KA2, SBF2, SLC25A23, SMAD7, SPEF2 SRPK1 TNFAIP1, TTC13, RGS2, RNF220, RNLS, RPL11, RYR1, SEMA4D, SERPING1, SMAD3^68^, SMAP2, SPINT1, TTLL11, WDFY2, WDR60, WHSC1L
COAD	Up: ANK1, CAMK2D, CBLB, CHD3, FAM124B, HIVEP2, MAP3K5, NHSL2, POLR3GL, PPP3CA, RASGRP4, RGS2, RPS6KA Down: BZRAP1, CDK8, CHEK2, HSF4, MAPKAPK
LUAD	Up: ASB2, CAMK2D, CCR6, CD226, CD247, IL18R1, IL2RB^66^, ITK, MAP3K5, NHSL2, PRKCH, RASGRP1, RASGRP4, RASSF5, RYR1, SEMA4D, SERPING1, ST8SIA1 WDFY Down: CCDC12, LSM4, MED29, MYL6B, PAF1, CX3CR1, FAM124B, GNA15^61^, IKZF3, IL13, RNF22
LUSC	Up: ARHGAP26^63^, CAMK2D, CCDC88C, CCR6, CD226^60^, IL2RB^66^, ITK, PPP3CA, PRKCH, RASGRP1, RASGRP4, RASSF5, SERPING1, SMAD7, SMAP2, XIRP Down: AGPAT5^67^, ETV5, HINT1, HSCB, MOBP, CD247^36^, CISH, GNA15^61^, HIVEP2, IKZF3, MYL6B, RNF220, SRPK

### Th1/Th2 balance in cancer survival

**Figure 5:**
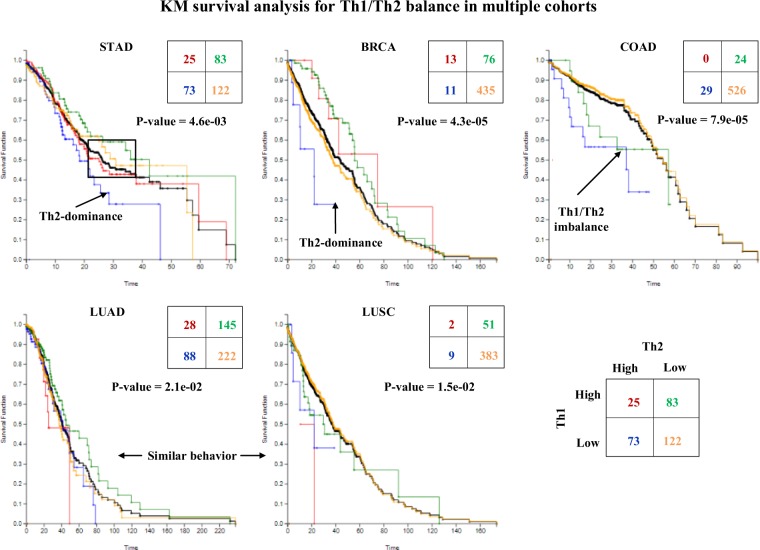
Th1/Th2 balancing can be explained from these results obtained from the KM analyses conducted using M scores. For example, Th1 and Th2 cells are known to eliminate tumor cell translation reducing its evasion effects on cancer cells and patients with highly activated immune pathways may have a better survival chance.

Figure 5 shows the result of analyzing four sub-groups obtained by high/low pathway scores between Th1/Th2 cell differentiation using M-score applied to the five cohorts. First, the KM analysis p-values obtained by comparing four subgroups are below 0.05 in all cases. In Table 3 we contrast these M based p-values with p-values obtained when R-score is used. It clearly shows that M based analysis outperforms R based analysis. Second, in every case, the sub-population labeled by low-low (i.e., the fourth quadrant in the contingency table) indicating repressed Th1 and Th2 cell differentiation represents the highest portion in each case (i.e., 90% for COAD, 86% for LUSC, 81% for BRCA, 46% for LUAD and 40% for STAD), consistent with a reduced effective immune response in cancers. Third, in both STAD and BRCA, patients with Th1 low and a Th2 high (namely, Th2 dominance) show a poorer prognosis, whereas those with Th1 dominance show a better prognosis. In COAD, patients with either Th2 dominance (red line) or Th1 dominance (green) have a poorer prognosis. In LUAD, Th2 dominance correlates with a poorer prognosis, whereas in LUSC, the opposite can be said. We note that this survival analysis excludes progression-free patients (e.g., patients who survived for more than 5 years). Overall, this analysis shows that evaluating the tumor immune response by pathway-based gene expression effectively stratifies patients into different risk groups, and suggests distinct roles of the Th1 and Th2 responses in different cancer types.

**Table 3:** R-based and M-based survival analysis in Th1/Th2 balance.

Study	KM P-value by R	KM P-value by M
STAD	2.3e-01	4.6e-03
BRCA	4.3e-02	4.3e-05
COAD	1.1e-02	7.9e-05
LUAD	5.5e-02	2.1e-02
LUSC	8.8e-03	1.5e-02

**Figure 6:**
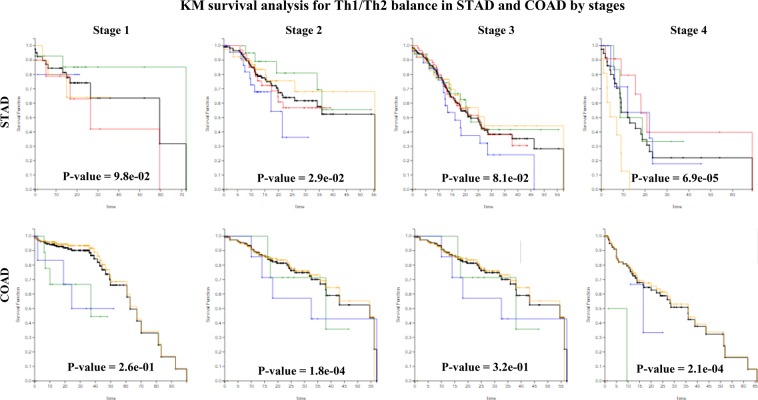
The results of studying the Th1/Th2 balancing using cancer stage data show meaningful outcomes in case of STAD and COAD. Same color coding was applied as in Fig. 5.

Figure 6 shows the analysis outcome of Th1/Th2 balance in which cancer stage is considered^14^. In this analysis, STAD and COAD cohorts are chosen because they show distinct effects of Th1 and Th2 responses on patient survival. In STAD, the Th2 association with a poorer prognosis is seen most clearly at Stage 2, which tends to gradually diminish as the cancer progresses^15,16^. A similar observation is made with COAD patients; Th1 and Th2 dominant colon cancers exhibit poorer prognoses at Stage 2, after which this trend becomes less clear. This analysis shows the importance of cancer stage in determining the possible impact of Th1 and Th2 responses in patient stratification.

### Mediating downregulation signal of BCL6 pathway

**Figure 7:**
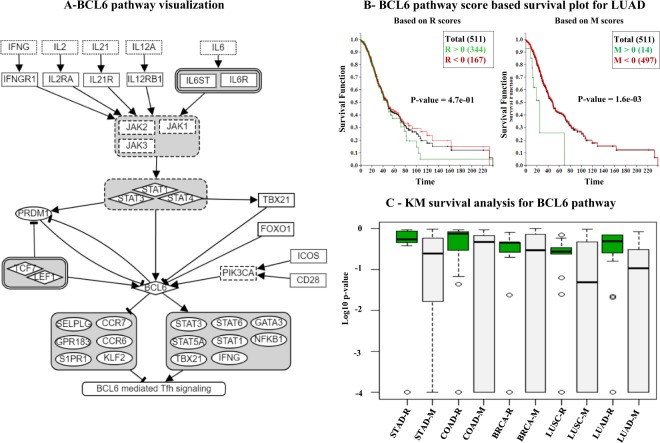
(**A**) Pathway visualization of BCL6 pathway. (**B**) Survival analysis based on the pathway scores for Lung Adenocarcinoma cancer with R and M thresholded at zero (e.g., *R* > 0*vs*.*R* < 0 and *M* > 0 *vs*. *M* < 0). (**C**) Cohort-based study for BCL6 pathway. Most of the studies have KM p-values smaller for *M* compared with *R*. Theistribution is shifted to bottom. The extended pathway outperforms the original one on all five cohorts in this KM survival analysis.

BCL6 is a transcriptional repressor being considered as a critical regulator of germinal centers where B cells are selected on production of high affinity antibodies^17^. The BCL6 pathway is shown in Fig. 7A in which its upstream path includes IFNG, JAK, and STAT. The upstream portion of transcription factor BCL6 has been adopted from Park *et al*.^18^ and its downstream portion including target genes such as STAT, GATA, CCR, NFKB1 and so on from Hatzi *et al*.^19^. Figure 7B shows the KM analysis outcome performed using LUAD cohort, demonstrating the improvement of using Up/Down target genes (i.e., R-score based p-value 0.47 vs. M-score based p-value 0.0016). Note that the red line is for the R > 0 group and the green line is for R < 0 group. Similarly, M > 0 and M < 0 are for the red/green groups respectively. Figure 7C shows the summary of all KM p-values produced by varying thresholds using a pair of box plots for each cohort. What is noticeable here is that M-score based KM analyses produce smaller p-values in all five cases, while in four cases (COAD, BRCA, LUSC and LUAD) some of M-score analyses produce very small p-values (less than 0.0001), suggesting the clear benefit of using BCL6 target genes in the analysis. Lastly, Table 4 is the comparison of KM analysis p-values for this BCL6 pathway. It repeats the pattern as in Table 3 that M based analysis outperforms R based analysis in all five cases.

**Table 4:** BCL6 pathway scores based survival analysis.

Study	Threshold *β*	KM P-value by R	KM P-value by M
STAD	0	4.7e-1	6.1e-02
BRCA	−0.5	3.4e-3	1.4e-3
COAD	−0.2	2.2e-2	3.9e-4
LUAD	0	4.7e-1	1.6e-3
LUSC	0.5	6.9e-1	2.6e-1

## Discussion

### Significance test for tailored pathway against decoy pathways

BioTarget is a pipelined bioinformatics system that allows scientists to identify a small number of testable Up/Down target genes of a pathway including key regulatory TFs. Here we focus on immune response pathways, specifically Th1 and Th2 differentiation, in which roles of TBX21 and GATA3 are firmly established. Generating the small number of target genes is like distilling or enriching from raw materials (ENCODE ChIP targets in our case identified for TBX21 and GATA3) into a small number which demonstrate a strong correlation with the upstream signals known to direct the differentiation pathways. One way to demonstrate the significance of our derived gene sets is to calculate the statistical significance of output genes. We carried out a decoy pathway analysis, an approach which is similar to what has been done in PARADIGM^6^. In Th1 cell differentiation pathway, which has been extended with Up/Down targets, a decoy pathway is created by replacing each gene in the pathway with a randomly selected one while the relationships established in the pathway are kept identical. A total of ten random decoy pathways were created to test against the tailored pathway for Th1 Cell Differentiation (Th1*). We used statistical tests with different criteria, which include TTest, Wilcoxon ranked sum test for difference and correlation. The outcome of this significance test is summarized in Table 5. We have a “significant” result if at least 2 of 3 criteria are statistically significant. For example, Random1 and Th1* (score distributions) relation has TTest and Wilcoxon p-values of 6.90E-13 and 6.42E-14 indicating both are significant and their correlation coefficient is 0.14. It means that these two probability distributions clearly have dissimilarity. In Table 5, we have all “significant” results.

**Table 5:** Significant tests for Th1 Cell Differentiation decoy pathways (Random i) and a tailored pathway for gastric cancer cohort (Th1*).

Pathway scores	TTest	Wilcoxon	Correlation
Random1 and Th1*	6.90E-13	6.42E-14	0.14
Random2 and Th1*	1.98E-26	2.95E-23	0.17
Random3 and Th1*	4.43E-18	5.87E-19	0.07
Random4 and Th1*	3.68E-05	1.36E-06	0.34
Random5 and Th1*	4.48E-21	9.67E-20	0.19
Random6 and Th1*	1.87E-22	2.11E-21	0.11
Random7 and Th1*	7.81E-33	9.94E-27	0.01
Random8 and Th1*	1.01E-25	6.65E-23	0.23
Random9 and Th1*	1.51E-10	8.21E-11	0.02
Random10 and Th1*	3.19E-20	2.63E-22	0.26

### Venn diagram analysis of Up/Down target genes among different cancer cohorts

**Figure 8:**
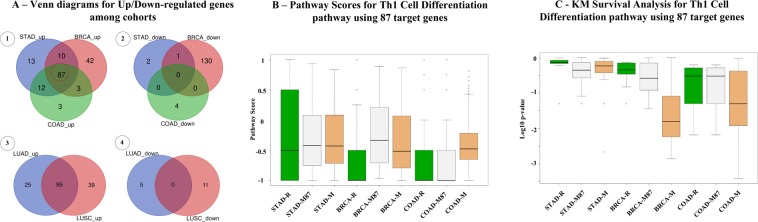
(**A**) Venn diagrams reveal significant overlaps among Up targets but little overlap among Down targets. (**B**) Box-plots for pathway scores for three cohorts demonstrate significant variance. R and M scores range from −1 to 1. (**C**) Comparing the outcomes of KM survival analysis suggests the strength of using M score for the pathway analysis.

After identification of Th1/Th2 target genes using BioTarget, one question concerns how many genes are common to all cancer types, and how many are cancer-type specific. Figure 8A shows the outcome of intersecting Up/Down target gene sets obtained from BioTarget when the threshold of correlation coefficient for the inclusion decision is fixed at 0.3 for positive correlation and −0.3 for negative correlation. Figure 8A1 shows the overlapping Up/Down target genes identified from using the three cancer type cohorts, STAD, BRCA and COAD, in which 87 genes (48%) of the entire Up genes identified are common among the three types whereas none such common gene is found among Down targets. Noticeable here is that a very large number (131) of Down targets are identified in BRCA alone. The figure also includes Venn diagrams of commonly identified genes of a different set of two lung cohorts, LUAD and LUSC, in Fig. 8A3,A4 with 99 overlapped genes (60%). In case of intersecting all five cohorts, commonly found Up/Down targets in all cases are 0 and 0 as shown later in Supplementary Information. In this section, venn diagram analysis for GATA3 Up/Down targets is also reported. Figure 8B,C describe how different are three cohorts when using the pathway with 87 common Up genes. M87 scores are M scores of pathways generated using only 87 common genes. In Fig. 8B, pathway scores are mostly negative that lead to suppressed stages of Th1 signals among three cohorts. In Fig. 8C, KM p-values for analysis which used regular M having the lowest p-values are shown. This observation supports the need of using “tailored” pathway for each cancer cohort with different context-dependent target genes. Another observation is that some gene identified as a Up target in one cancer type is also identified as a Down target in another cancer type (e.g., SMAP2 as discussed below).

### Biological significance of identified cancer-type specific target genes

As shown in Table 1, many genes identified as TBX21 target genes using different cancer cohorts have been annotated as cancer related or immune related in the literature. This finding suggests that BioTarget is capable of producing statistically and biologically significant target genes in a context-specific manner. Here are some examples demonstrating such capability of BioTarget. ILF2 identified as a Down target for LUAD in Table 1 has been found to functionally affect various cancer types. According to Ni *et al*.^20^, expression level of ILF2 is up-regulated in non-small cell lung cancer (NSCLC), and knockdown of ILF2 inhibits the cell proliferation and cell-cycle progression. Another example is LAX1, which is known to be an essential immune-relevant gene encoding membrane-associated adaptor protein on B and T lymphocytes. In Table 1, LAX1 is identified and categorized into an Up target gene in STAD. Zhu *et al*.^21^ showed that LAX1 can control Ras-MAPK activation, Ca++ flux and nuclear factor of activated T cells (NFAT) activation.

BioTarget is also capable of identifying target genes whose role in cancer or the immune response has not been previously identified. For example, SLC35B2 gene encodes solute carrier transporter protein that transports sulfate from cytosol to Golgi and is considered an important step in the syntheses of glycoprotein and glycolipid as reported by Kamiyama *et al*.^22^. In Table 1, SLC35B2 is reported as a down-regulated target gene in LUAD cancer. This finding suggests the suppressing role of SLC35B2 resulting in a functional perturbation of glycolipid synthesis and consequently indirectly impacting the immune response of LUAD patients. Another interesting target gene reported in the table is ITGAL, an integrin-family cell adhesion molecule. Integrins are known to interact with intercellular adhesion molecules 1–3 (ICAM1-3) and mediate the cellular recognition and migration of leukocytes according to Corbi *et al*.^23^. Although leukocyte adhesion and migration are closely-related to humoral immune response, function of ITGAL in cancer immune-oncology remains unclear. Here in Table 1, ITGAL is identified by BioTarget as an Up target gene in COAD, LUAD and LUSC.

For the GATA3-mediated Th2 differentiation pathway, similar argument can be made that BioTarget can determine Up/Down target genes of this pathway. According to Klinke 2014^24^, CD247 encodes the T-cell receptor zeta, which responds to the T-cell mediated type 1 cytotoxic immune signaling by stimulating expression of TBX21 and suppressing expression of GATA3. As shown in Table 2, CD247 is identified and categorized to as a GATA3 Up target gene in STAD, BRCA and LUAD. Another example demonstrating the capability of target discovery is SMAP2, included as an Up target for STAD, BRCA and LUSC. As reported by Natsume *et al*.^25^, SMAP2 gene encodes stromal membrane-associated GTPase-activating protein 2, which is known to activate GTPase and interact with clathrin. Although specific functions of SMAP2 in cancer or immune are yet to be known, BioTarget seems to suggest that the altered expression of SMAP2 gene in GATA3 may mediate Th2 differentiation pathway in a cancer type specific manner, that is, through up-regulation in LUSC but through down-regulation in STAD and BRCA.

### Pathway visualization to improve comprehensibility

The goal of this part is to demonstrate how to incorporate heuristic method into a visualization tool to control pathway components. Each pathway could combine multiple pathways and can exponentially grow unless the complexity of the network is controlled. The existing web-based interactive gene network programs such as PCViz by Cerami *et al*.^26^, GeneMania by Warde *et al*.^27^ are powerful but do not address the data reduction issue.

**Figure 9:**
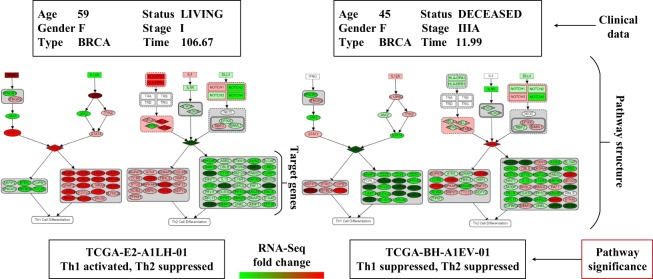
Data visualization of simplified Th1 Cell Differentiation pathway of two patients in different stages of breast cancer. A typical pathway includes multiple components including ligands, receptor, kinase, transcription factors, target genes, and biological process as mentioned in Fig. 1. RNA-seq fold change is ranging from −5 to 5. Each pathway component is assigned with RNA-seq fold change (tumor vs. control log2 ratio). For example, TBX21 gene of TCGA-E2-A1LH-01 has RNA-seq fold change of 2.18, presented in red color. Clinical and other information of subjects have been added to examine the model. The pathway’s activation/suppression status is more clear from the color coding of the genes appearing as TF targets.

In Figs 1 and 9, upstream of Th1/Th2 Cell Differentiation pathway is a combined diagram which is created by literature and other sources such as KEGG and WikiPathways. RNA-seq transcriptome data of each gene is sprayed in the diagram. Two extreme cases of Th1/Th2 Cell Differentiation have been selected. Clinical data shows the great association of pathway with the prognosis. The mapping process from transcriptome data of all genes of each patient and component names (in the pathways) has been completed. Our approach was to introduce a set of data reduction heuristics for vast majority of data reduction which is followed by minimal user rendering of displayed objects (particularly the layout). Pathway visualization helps us acquire comprehensibility effectively. In Fig. 9, the signals of Th1 and Th2 Cell Differentiation are greatly associated with clinical data as the result was shown in the previous section. Patient with ID of TCGA-E2-A1LH clearly has Th1 activated and Th2 suppressed with all target genes working consistently to support the concept. In contradiction, patient with ID of TCGA-BH-A1EV has both Th1/Th2 suppressed with poor prognosis as a result. When coordinating a transcriptome profile of patient with respect to the context of a cancer cohort and target genes of TFs, scientists can validate the result of target genes and how they interact with TFs and other components in the pathways with transcriptional targets.

## Methods

### Identifying potential target genes from ENCODE ChIP-seq data

**Figure 10:**
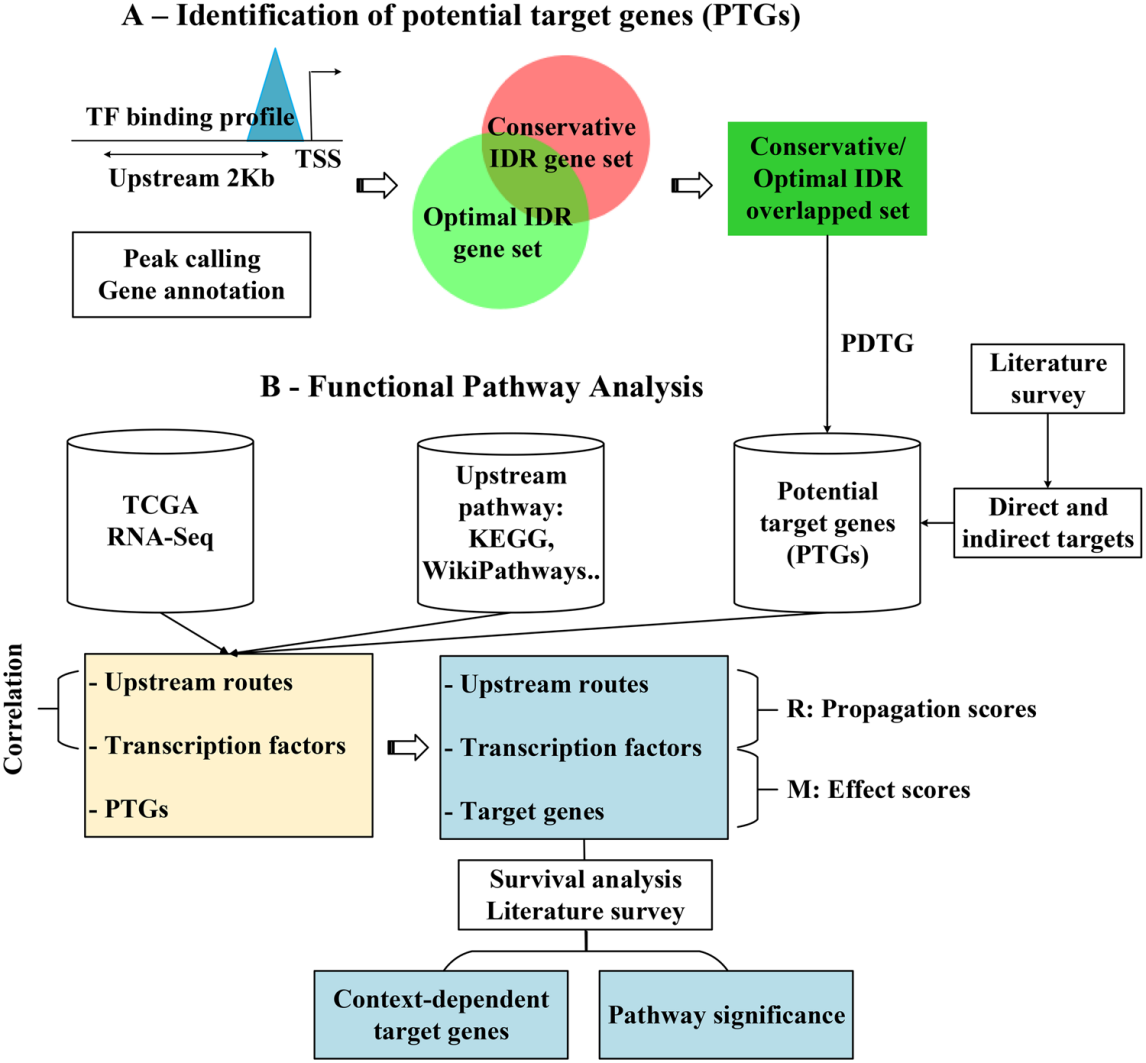
(**A**) ChIP-seq peaks located near 2Kb upstream distance from transcription start site are suggestive of direct target genes of a transcription factor with high probability. Direct and indirect targets can be added from literature survey. (**B**) Pipeline for identifying TF target genes for a pathway and extending the pathway with the identified up-regulated and down-regulated genes. The significance of the extended pathway is assessed by performing KM survival analyses and literature survey.

The overview of the BioTarget data processing pipeline is outlined in Fig. 10. The initial step, labelled Part A of Fig. 10 aims to identify potential direct target genes of a transcription factor using the ChIP-Seq data sets available in ENCODE in which the quality of peaks has been determined by Irreproducible Discovery Rate (IDR), measuring consistency between replicates produced from high-throughput ChIP-seq experiments^28^. ENCODE peaks are published with IDR, which could be used as a cutoff to include only a small subset of the identified peaks for downstream analysis. The first step of this downstream analysis is using the open source Bioconductor package ChIPpeakAnno which maps ChIP-seq peaks into candidate target genes of the concerned transcription factor (Zhu *et al*.^29^). We used human assembly reference GRCh38 for gene annotation and we opted to use “2Kb” distance from binding site to a gene’s transcription start site (TSS). The choice of “2Kb” can be modified to a smaller or bigger number depending on the intent to produce a larger or smaller size of what we call “Potential Direct Target Genes (PDTG)” as the input for Part B as shown in Fig. 10. Another method to control the size of PDTG is which IDR value is used. For example, since ENCODE produces Optimal and conservative IDR sets, either conservative IDR thresholded peaks, or optimal IDR thresholded peaks, or any Boolean combination between the two could be used to form PDTG. We chose to intersect Conservative and Optimal IDR gene sets in which TBX21 has 52%, GATA3 has 14%, and BCL6 has 66% in common as shown in Supplementary Information. Yet another way to prepare PDTG is to consider source cell lines of the ENCODE published peaks^28^, for example, if the cell line used for the peak generation is from GM12878 immortalized B-Lymphocyte, MCF-7 breast cancer, HepG2 human liver cancer, or K562 immortalized myelogenous leukemia. We show the outcome of our assessment on how much overlap between Conservative and Optimal IDR gene sets is observed when our PDTG generation method is applied to six transcription factors: TBX21, GATA3, BCL6, IRF5, PAX5, and STAT1 in Supplementary Information. These transcription factors are known for their key roles in immune cell development^30^. Significant overlap was observed in some cases but not in all cases, suggesting the need of careful consideration during PDTG generation. Lastly, as shown in Fig. 10 PDTG can be augmented by including indirect transcription factor targets which can be typically gathered through literature survey. We label the initial PDTG augmented with the optionally added indirect target genes “Potential Target Genes (PTG)”.

### Determining downstream targets of a transcription factor from PTG

Next is to “distill” PTG to produce a far smaller subset of genes which are more likely legitimate targets of the concerned pathway. Given a transcription factor *f*, let its true downstream be denoted by *T*(*f*). *T*(*f*) may vary depending on the context (i.e., time and space of a biological condition) and finding *T*(*f*) is known non-trivial (Nakamura *et al*.^31^). Our strategy is using a route-based scoring scheme which transforms the degree of overly activated or suppressed pathway status into scores^8,9^ and uses the scores to compute how likely a member of PTG should be included in *T*(*f*). Our scoring scheme is made up of two parts, namely, route propagation score (R) and effect score (M) as shown in Fig. 10, for each recognizable route of the pathway defined for biological process.

#### Computing route propagation score (R)

R signifies the strength of a particular route(s) potentially responsible for the transcriptome level change of *f*, while M estimates the activation/suppression strength of the pathway toward the biological process the pathway is defined for. Propagation score of a route *j* in a pathway *P* denoted by ${R}_{P}^{j}$ is defined as follows.

1 $${R}_{P}^{j}=\frac{{\sum }_{i=1}^{{n}_{j}}I(v({g}_{t},{g}_{i},{r}_{j}) > 0)-{\sum }_{i=1}^{{n}_{j}}I(v({g}_{t},{g}_{i},{r}_{j}) < 0)}{{n}_{j}}\times (1-FD{R}_{j})$$

where

2 $$FD{R}_{j}=\frac{{n}_{g}\times {t}_{pr}}{{\sum }_{i=1}^{{n}_{g}}I({p}_{ij} < {t}_{pr})}$$

here *r*_*j*_ is a route (1 ≤ *j* ≤ *n*_*j*_) where *n*_*j*_ is the number of routes in the pathway *P*; *g*_*i*_ and *g*_*t*_ are a gene and a transcription factor in the route *r*_*j*_, respectively; *n*_*g*_ is the number of genes in the route; *I*(·) is an indicator function of which its value is 1 if the condition meets or 0 otherwise; *v*(*g*_*t*_, *g*_*i*_, *r*_*j*_) is the consistency value of the gene *g*_*i*_ in the route *r*_*j*_ with respect to the transcription factor *g*_*t*_. Let the gene *g*_*i*_ activate *g*_*t*_, then *p*_*ij*_ is the p-value assigned to *g*_*i*_ in the route *r*_*j*_ and ${\sum }_{i=1}^{{n}_{g}}I({p}_{ij} < {t}_{pr})$ is the total number of genes which have lower p-values than a pre-determined p-value threshold *t*_*pr*_. The p-value of *g*_*i*_ in *r*_*j*_ is calculated by the area beyond the observed data point of the probability density function (PDF) of the null hypothesis (${\int }_{{g}_{i}}^{\infty }h(s)ds$, where *h*(*s*) is the PDF of the gene values of the population). Here *g*_*i*_ and *g*_*t*_ are log2 ratio values and they can have positive values when the test value is greater than the control value, or negative values when the test value is less than the control value. If *g*_*i*_ and *g*_*t*_ have the same sign, *v*(*g*_*t*_, *g*_*i*_, *r*_*j*_) = 1, and if the signs are opposite, *v*(*g*_*t*_, *g*_*i*_, *r*_*j*_) = −1. In the case that *g*_*i*_ inhibits *g*_*t*_, the *v*(*g*_*t*_, *g*_*i*_, *r*_*j*_) = −1 if the signs of *g*_*i*_ and the *g*_*t*_ are the same, or *v*(*g*_*t*_, *g*_*i*_, *r*_*j*_) = 1. In Equation 1, for each gene *i* in route *j*, ${\sum }_{i=1}^{{n}_{j}}I(v({g}_{t},{g}_{i},{r}_{j}) > 0)$ is the number of genes which are consistent with the *TF* expression value, while ${\sum }_{i=1}^{{n}_{j}}I(v({g}_{t},{g}_{i},{r}_{j}) < 0)$ is the number of genes which are not consistent wi_*t*_h *g*_*t*_.

The p-value is a probability that the observed data point (sample) is how different from the data set of null hypothesis *H*_0_ (population)^32^. It is calculated as the total area of the PDF of *H*_0_ beyond the observed data point. If *Y* is a random variable of observation and *y* is an actual observed data point, p-value is *Pr*(*Y* ≥ *y*|*H*_0_), *Pr*(*Y* ≤ *y*|*H*_0_), or 2 × *min*(*Pr*(*Y* ≥ *y*|*H*_0_), *Pr*(*Y* ≤ *y*|*H*_0_)) in the cases of up-regulated genes, down-regulated genes, up/down regulated genes, respectively.

#### Computing effect score (M)

Effect score of a pathway *P* denoted by *M*_*P*_ is defined as follows

3 $${M}_{P}=\frac{({\sum }_{i=1}^{{n}_{m}}I(v({g}_{t},{g}_{i},BP) > 0)-{\sum }_{i=1}^{{n}_{m}}I(v({g}_{t},{g}_{i},BP) < 0)}{{n}_{m}}\times (1-FD{R}_{m})$$

where

4 $$FD{R}_{m}=\frac{{n}_{m}\times {t}_{pm}}{{\sum }_{i=1}^{{n}_{m}}I({p}_{i} < {t}_{pm})}$$

Similar to ${R}_{P}^{j}$ in Equation 1, for each target gene *g*_*i*_, ${\sum }_{i=1}^{{n}_{m}}I(v({g}_{t},{g}_{i},BP) > 0)$ is the number of target genes which consistent with Biological process *BP*, while ${\sum }_{i=1}^{{n}_{m}}I(v({g}_{t},{g}_{i},BP) < 0)$ is the total number of target genes which are not consistent with *BP*. *BP* is estimated by the signal of *TF*. *n*_*m*_ is the number of target genes in downstream. ${\sum }_{i=1}^{{n}_{m}}I({p}_{i} < {t}_{pm})$ is the number of genes of which p-values are less than predetermined p-value threshold *t*_*pm*_. A route is traversing from *BP* backward to *TF* through up-regulated genes and down-regulated genes of downstream targets. Both scores of ${R}_{P}^{j}$ and for *M*_*P*_ are leveraging biological process evaluation. When *A*_*k*_ is the number of consistent and significant genes with p-values less than threshold, *A*_*k*_ × (1 − *FDR*) will be a number of truly consistent and significant genes. Effect score *M*_*P*_ of a patient *P*_*k*_ is calculated as ${M}_{P}={A}_{k}\times \frac{1-FDR}{n+m}$, where *n* and *m* are the number of down-regulated genes, and up-regulated genes, respectively.

#### Selecting Up and Down target genes

Choosing Up and Down target genes is achieved in two steps. The first step is to identify two sub-cohorts of the original cohort *C* of the subjects whose transcriptome patterns can be considered conveying some strong regulatory signals in one way or the other for the pathway *P* under consideration. Those demonstrating strong activation signals are grouped into the sub-cohort, say *C*_*u*_, and those demonstrating strong suppression signals are grouped into the sub-cohort, say *C*_*d*_. The R score computed for each subject of the cohort *C* and some preset threshold values are used to make the membership decision. But the FDR introduced in Equation 2 can further control the size of *C*_*u*_ and *C*_*d*_. The second step is to use transcriptome patterns included in *C*_*u*_ and *C*_*d*_ as references in deciding if a gene *g*_*i*_ in PTG should be classified as an Up or Down target of the transcription factor *g*_*t*_ in *P*. For this step Pearson correlation coefficients are calculated between *g*_*t*_ and *g*_*i*_ for the transcriptome patterns included in *C*_*u*_. and *C*_*d*_.. Some preset threshold values for the Pearson correlation coefficient are used to determine if *g*_*i*_. should be included in the Up target group for, say *G*_*u*_ or the Down target group, say *G*_*d*_. In addition the FDR introduced in Equation 4 can further control the size of *G*_*u*_ and *G*_*d*_. The pathway is extended with two types of target genes, the Up targets in *G*_*u*_. and the Down targets in*g*_*t*_ *G*_*d*_. Below in Table 6 we illustrate these notations and the basic concept applied to extending the Th1 Cell Differentiation pathway with five cancer cohorts, STAD, BRCA, COAD, LUAD and LUSC.

**Table 6:** Extending Th1 Cell Differentiation pathway with estimated targets of TBX21.

Study		Cohort Size		Thresholds		# Genes		Correlation	
ID	Name	C	C′	${{\boldsymbol{t}}}_{{{\boldsymbol{g}}}_{{\boldsymbol{u}}}}$	${{\boldsymbol{t}}}_{{{\boldsymbol{g}}}_{{\boldsymbol{d}}}}$	#*G*_*u*_	#*G*_*d*_	CorC	CorC′
STAD	Stomach Adenocarcinoma	414	183	0.7	−0.3	30	17	0.657	0.787
BRCA	Breast Carcinoma	1101	642	0.7	−0.5	22	7	0.718	0.824
COAD	Colorectal Adenocarcinoma	614	332	0.7	−0.3	4	17	0.522	0.743
LUAD	Lung Adenocarcinoma	507	175	0.4	−0.3	10	5	0.584	0.78
LUSC	Lung Squamous Cell Carcinoma	490	195	0.7	−0.3	35	11	0.493	0.661

#### Algorithm

The detailed procedure to “distill” target genes based on ${R}_{P}^{j}$ and *M*_*P*_ scores of the pathway *P* is given below.

Input: Pathway *P*, cohort $C({C}_{1},{C}_{2},\ldots ,{C}_{{n}_{c}})$, and candidate target gene set *G*.

Output: Extended pathway *P*′, possible target gene set *G*′ for the context defined by the cohort *C*.

1. *Compute R score:* Calculate ${R}_{P}^{j}$ scores with Equation 1.
2. *Choose two sub-cohorts and combine them:* From the values of ${R}_{P}^{j}$ calculated for each subject *C*_*q*_, 1 ≤ *q* ≤ *n*_*C*_, determine the two sub-cohorts *C*_*u*_ and *C*_*d*_ as follows.
  $$\begin{array}{rcl}{C}_{u} & = & \{{C}_{q}|{R}_{P}^{j}({C}_{q}) > {t}_{cu}\}\\ {C}_{d} & = & \{{C}_{q}|{R}_{P}^{j}({C}_{q}) < {t}_{cd}\}\\ C^{\prime}  & = & {C}_{u}\cup {C}_{d}\end{array}$$
  *C*_*u*_ and *C*_*d*_ include only the subjects whose ${R}_{P}^{j}$ values are higher or lower than the two preset thresholds *t*_*cu*_ and *t*_*cd*_, respectively. Both are combined into *C*′, called the selected cohort.
3. *Compute Pearson correlation coefficients:* Calculate Pearson correlation coefficients (${\rho }_{P}^{i}$) between *g*_*t*_ in the pathway *P* and each candidate target gene (*g*_*i*_) using the selected cohort *C*′ ($C^{\prime} ={C}_{u}\cup {C}_{d}$). A candidate target gene set (*G*) can be collected from the literature or ChIP-seq experiments.
  $${\rho }_{P}^{i}({g}_{t},{g}_{i},{C}_{u},{C}_{d})=\frac{{\sum }_{k=1}^{{n}_{C^{\prime} }}({g}_{i,k}-{\bar{g}}_{i})({g}_{t,k}-{\bar{g}}_{t})}{{\sum }_{k=1}^{{n}_{C^{\prime} }}({g}_{i,k}-{{\bar{g}}_{i}}^{2})\times {\sum }_{k=1}^{{n}_{C^{\prime} }}({g}_{t,k}-{{\bar{g}}_{t}}^{2})}$$
  where *n*_*C*′_ is the number of the selected cohort *C*′. ${\bar{g}}_{i}\,{\rm{and}}\,{\bar{g}}_{t}$ are the mean values of *g*_*i*_ and *g*_*t*_ of the selected cohort *C*′, respectively. With 1 ≤ *k* ≤ *n*_*C*′_, *g*_*i*,*k*_ and *g*_*t*,*k*_ are, respectively, candidate target gene *g*_*i*_ and transcription factor *g*_*t*_ appearing in the *k*-th patient transcriptome data set included in the selected cohort *C*′.
4. *Select two subsets from potential target genes:* Select the subset *G*_*u*_ of *G* such that ${\rho }_{P}^{i}({g}_{t},{g}_{i},{C}_{u},{C}_{d})$ is greater than up-gene threshold (${t}_{{g}_{u}}$) and subset *G*_*d*_ of *G* such that ${\rho }_{P}^{i}({g}_{t},{g}_{i},{C}_{u},{C}_{d})$ is less than down-gene threshold (${t}_{{g}_{d}}$). Various thresholding strategy can be applied, such as, customized, quantile or absolute thresholding.
  $$\begin{array}{rcl}{G}_{u} & = & \{{g}_{i}|{\rho }_{P}^{i}({g}_{t},{g}_{i},{C}_{u},{C}_{d}) > {t}_{{g}_{u}}\}\\ {G}_{d} & = & \{{g}_{i}|{\rho }_{P}^{i}({g}_{t},{g}_{i},{C}_{u},{C}_{d}) < {t}_{{g}_{d}}\}\\ G^{\prime}  & = & {G}_{u}\cup {G}_{d}\end{array}$$
5. *Update the Pathway:* Update pathway *P* into *P*′ with *G*_*u*_ and *G*_*d*_.
6. *Compute M Scores:* Calculate *M*_*P*′_ scores using Equation 3.

### Evaluating the quality of the extended pathway

Once the pathway extension procedure is completed with some choices of parameters (i.e., threshold values and FDRs), an evaluation is performed. One particular evaluation method available when dealing with cancer data sets is the KM survival estimate method. We test if the addition of two types, *G*_*u*_ and *G*_*d*_, as TF targets in the pathway improves p-value obtainable from the KM survival analysis in a way the functional role of the concerned pathway can be meaningfully plained. The first step is to partition the cohort *C* into several sub-cohorts using the calculated *M*_*P*′_ scores. For example, for a binary sub-grouping, some threshold value is used to partition the cohort *C* into one for having high *M*_*P*′_ scores and one for having low *M*_*P*′_ scores and then use them to calculate KM survival rate and p-value and visualize the outcome. Another meth for the evaluation is calculating two sets of correlation between *R* scores and *M* scores (the first one *CorC* obtained by applying to the original cohort *C* and the second one *CorC*′ obtained by applying to the trimmed cohort *C*′) and compare them. The correlation coefficient obtained from *C*′ should be bigger than the one obtained from *C*., as illustrated in Table 6. This aluation process may repeat with different choices of parameters to find the trend of the solution values from each iteration.

## Conclusion

BioTarget is a new tool that uses “similar kind” transcriptome datasets together with ChIP-seq data to extend existing curated signaling pathways by incorporating TF activities. To demonstrate such pathway extension is possible, we took advantage of the publicly available TCGA datasets of different cancer types and the community-curated ENCODE ChIP-seq data. We treat each cancer type data (transcriptome and clinical, i.e., survival data) to form a context, and examine if and how known pathways can be extended. To demonstrate the feasibility of this approach, we used the BioTarget to quantify the activities of key T cell transcription factors associated with Th1 and Th2 cells. Interestingly, we found that different sets of TF downstream targets are discovered for different cancer types, together with a number of common target genes. We were able to use the extended pathways to stratify cancer patients into risk categories. Patient stratification by this method showed a number of cancer-type specific effects of the immune response, with a notably poor prognosis for patients with high Th2 scores for stomach breast and colon cancers. Using this tool, we also discovered that cancers scoring higher for BCL6 activity, a transcription factor expressed by T-fh cells, have a significantly better prognosis. Our data support the usefulness of the BioTarget tool for evaluating/scoring signaling pathway activity, for identifying context-specific biomarkers associated with cell signaling pathways, and for discovering TF target genes that underlie the changes in phenotype associated with these pathways.

BioTarget is a small step toward the new opportunity to extend existing signaling pathways with newly available data. There are many issues to be resolved. For example, the current implementation of BioTarget does not handle co-regulation by multiple co-factor TFs. TFs also act on non-coding regions, and how to incorporate TF regulation on non-coding regions poses an exciting challenge. We hope that this work lays the groundwork for a new way to extend pathways by systematically mining datasets with a context.

## Supplementary information

Supplementary info
