# Supplementary material for: BioTarget: A Computational Framework Identifying Cancer Type Specific Transcriptional Targets of Immune Response Pathways
Source: Sci Rep. 2019 Jun 21;9:9029. doi: 10.1038/s41598-019-45304-x (PMC6588588; doi:10.1038/s41598-019-45304-x)
Supplement: Supplementary file 1 — Supplementary info [file 41598_2019_45304_MOESM1_ESM.pdf]

BioTarget: A Computational Framework Identifying Cancer Type Specific Transcriptional Targets of Immune Response Pathways

Tham H. Hoang[1,*], Yue Zhao[1], Yiu Lam[1], Stephanie Piekos[2], Yueh-Chiang Han[3], Cameron Reilly[3], Pujan Joshi[1], Seung-Hyun Hong[1], Chang Ohk Sung[4], Charles Giardina[3], and Dong-Guk Shin[1,*]

1 Supplementary information

1.1 Potential direct target genes of key transcription factors in immune cell development

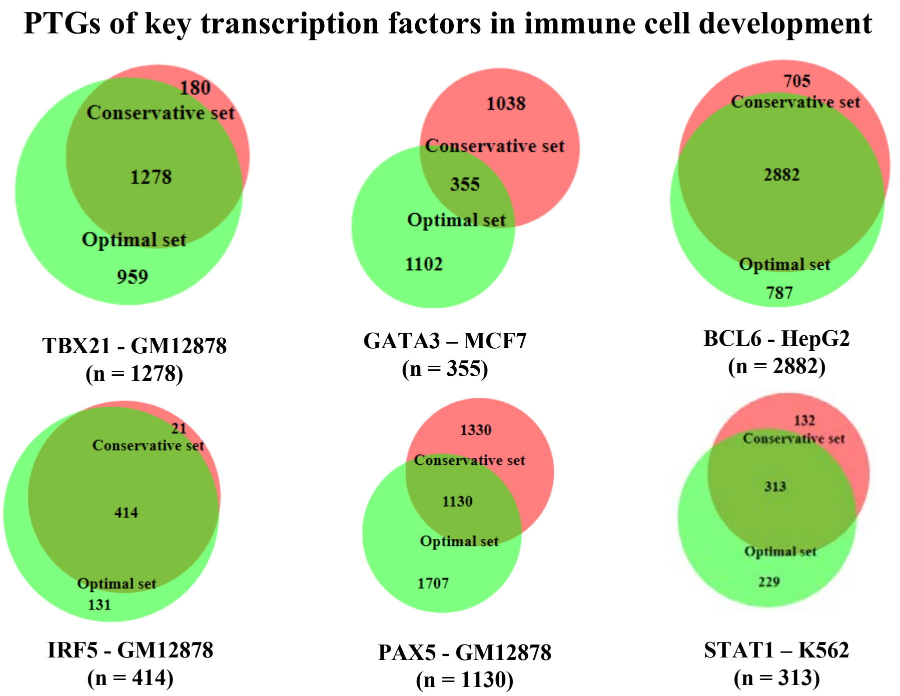

Figure 1: Identifying potential direct target genes for the six immune cell development related transcription factors identified by Smale et al. 2014 [1] by using two sets (Optimal set and Conservative set) reported by ENCODE. In the BioTarget analysis, the overlapped portions for TBX21, GATA3 and BCL6 are used. The cell lines used in the ENCODE ChIP-seq experiments are noted next to the transcription factor name.

1.2 A case study on transcription factor STAT3

STAT3 is a known transcription factor in mediated insulin-like growth factor (IGF) pathway. This pathway regulates organ development, growth, and survival by promoting cell proliferation and suppressing apoptosis. The IGF signaling is also involved in tumor growth, cancer progression, and metastasis. Targeting different components of the IGF signaling pathway is being attempted to develop novel cancer therapies. We used STAT3, a major component of the IGF pathway, to demonstrate the importance of "bringing in extra potential target genes known in the literature in addition to PDTG obtainable from ENCODE for the pathway target prediction. Total 81 target genes of STAT3 were identified from literature survey in coagulation, apoptosis, immunity, cell signaling, and interferon signaling. The procedure described in **??**A was used to identify 219 potential direct target genes (PDTG) of STAT3 from ENCODE cell line GM12878 by setting FDR at 0.05 and Log-rank test p-value at 0.05 using the filtering option available in BioTarget. Total 290 (= 81 + 219) PTG were used for the BioTarget analysis for the STAD cohort, which produced 11 Up genes and 12 Down genes. Among the UP target genes, 4 are from the survey set and 7 from the ENCODE set. Among the Down target genes, all 12 down genes are from the ENCODE set. Three different KM survival analyses were performed on three different combinations from the predicted targets, one including only those from ENCODE, one including only those from the survey and one including all. The p-values obtained were, respectively, 0.084, 0.46 and 0.044, suggesting that adding the genes from the literature survey can improve the prediction. One note for the process of adding the genes from literature survey is that it may add "indirect targets of the concerned transcription factor, although some published articles may list only

direct targets predicted from ChIP-seq experiments. Whether to use indirect targets for the analysis should be the use choice of the tool.

1.3 Transcription factors and pathways are analyzed by BioTarget pipeline

Table 1: Available sets of upstream and target genes of 16 transcription factors from ENCODE ChIP-seq datasets and other sources.

ID	Transcription factor	#Potential target genes	Pathway involved
1	BCL6	2882	BCL6
2	CREB1	420	MAPK
3	CREBBP	1625	Wnt
4	FOS	648	MAPK
5	FOXO3	197	MAPK
6	GATA3	355	Th2 Cell Differentiation
7	IRF5	414	Toll-like receptor
8	MYC	198	MAPK
9	PAX5	1130	Transcriptional misregulation in cancer
10	SPI1	125	Transcriptional misregulation in cancer
11	STAT1	313	Th1, Th2, and Th17 cell differentiation
12	STAT2	250	Jak-STAT
13	TBX21	1275	Th1 Cell Differentiation
14	TCF7	274	Wnt
15	TCFL2	389	Wnt
16	STAT3	219	IGF

1.4 Correlation analysis outcomes of representative example Up/Down targets of TBX21

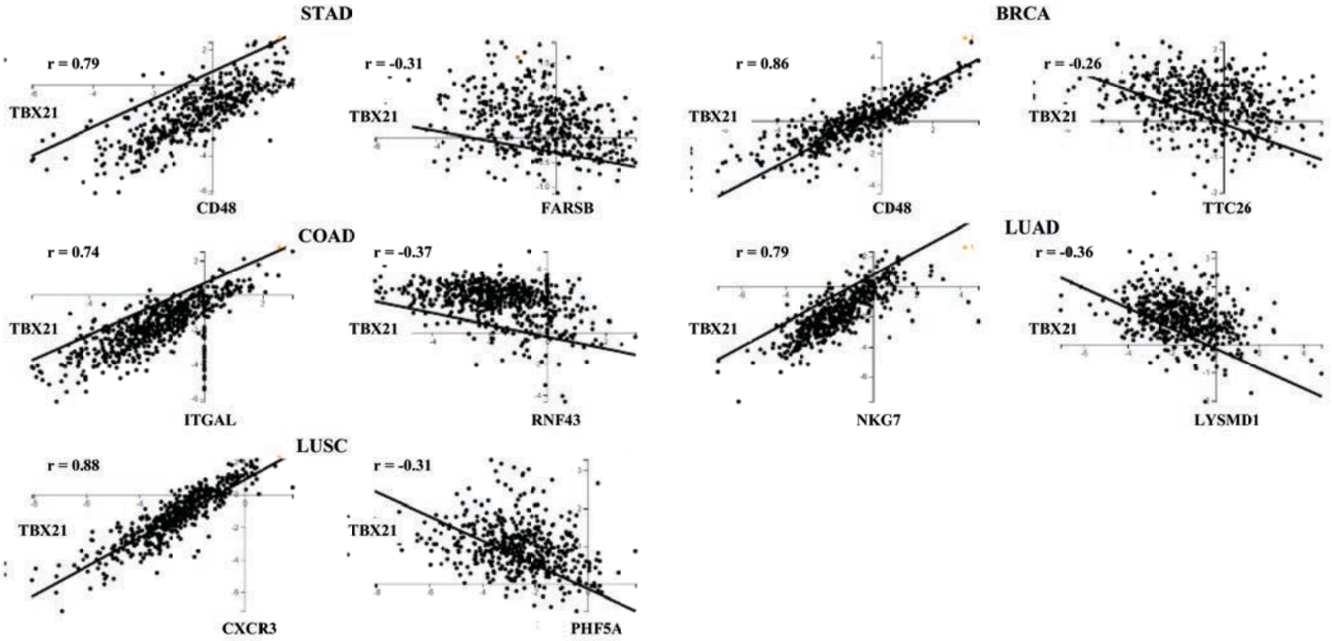

Figure 2: Correlation between TBX21 and UP/Down target genes of five cohorts.

1.5 Venn diagrams for five cancer cohorts comparing Up/Down targets of TBX21

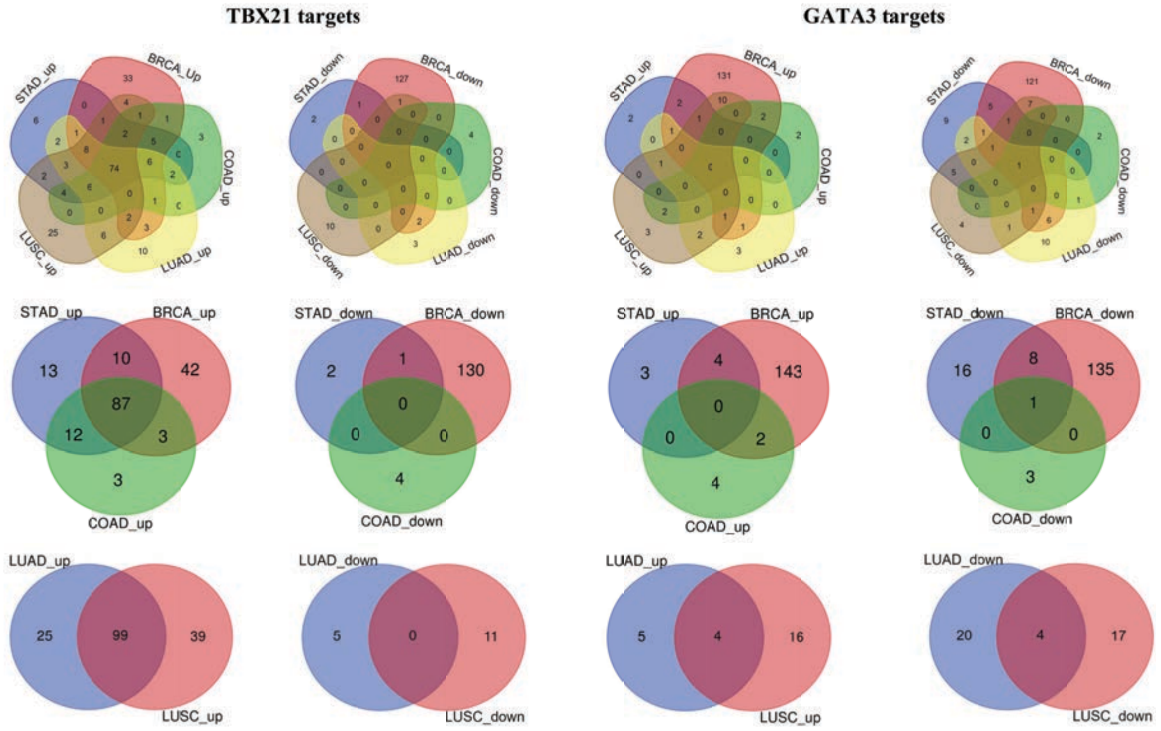

Figure 3: Down targets of TBX21 have no common in five cohorts.

References

[1] Stephen T Smale. Transcriptional regulation in the immune system: a status report. *Trends in immunology*, 35(5):190–194, 2014.
